# Common Data Elements to Facilitate Sharing and Re-use of Participant-Level Data: Assessment of Psychiatric Comorbidity Across Brain Disorders

**DOI:** 10.3389/fpsyt.2022.816465

**Published:** 2022-02-07

**Authors:** Anthony L. Vaccarino, Derek Beaton, Sandra E. Black, Pierre Blier, Farnak Farzan, Elizabeth Finger, Jane A. Foster, Morris Freedman, Benicio N. Frey, Susan Gilbert Evans, Keith Ho, Mojib Javadi, Sidney H. Kennedy, Raymond W. Lam, Anthony E. Lang, Bianca Lasalandra, Sara Latour, Mario Masellis, Roumen V. Milev, Daniel J. Müller, Douglas P. Munoz, Sagar V. Parikh, Franca Placenza, Susan Rotzinger, Claudio N. Soares, Alana Sparks, Stephen C. Strother, Richard H. Swartz, Brian Tan, Maria Carmela Tartaglia, Valerie H. Taylor, Elizabeth Theriault, Gustavo Turecki, Rudolf Uher, Lorne Zinman, Kenneth R. Evans

**Affiliations:** ^1^Indoc Research, Toronto, ON, Canada; ^2^Data Science and Advanced Analytics, St. Michael's Hospital, Unity Health Toronto, Toronto, ON, Canada; ^3^Rotman Research Institute, Baycrest Health Sciences, Toronto, ON, Canada; ^4^Hurvitz Brain Sciences Research Program, Dr. Sandra Black Centre for Brain Resilience and Recovery, Sunnybrook Research Institute, University of Toronto, Toronto, ON, Canada; ^5^Department of Medicine (Neurology), Sunnybrook Health Sciences Centre, University of Toronto, Toronto, ON, Canada; ^6^Mood Disorders Research Unit, University of Ottawa Institute of Mental Health Research, Ottawa, ON, Canada; ^7^School of Mechatronic Systems Engineering, Simon Fraser University, Surrey, BC, Canada; ^8^Department of Clinical Neurological Sciences, Schulich School of Medicine and Dentistry, University of Western Ontario, London, ON, Canada; ^9^Department of Psychiatry and Behavioural Neurosciences, McMaster University, Hamilton, ON, Canada; ^10^Mood Disorders Program and Women's Health Concerns Clinic, St. Joseph's Healthcare, Hamilton, ON, Canada; ^11^Department of Psychiatry, University Health Network, Toronto, ON, Canada; ^12^Department of Psychiatry, University of Toronto, Toronto, ON, Canada; ^13^Department of Psychiatry, University of British Columbia, Vancouver, BC, Canada; ^14^Division of Neurology, Department of Medicine, University of Toronto, Toronto, ON, Canada; ^15^Edmond J. Safra Program in Parkinson's Disease, University Health Network, Toronto, ON, Canada; ^16^Departments of Psychiatry and Psychology, Queen's University, Providence Care, Kingston, ON, Canada; ^17^Centre for Addiction and Mental Health, Campbell Family Mental Health Research Institute, Toronto, ON, Canada; ^18^Centre for Neuroscience Studies, Queen's University, Kingston, ON, Canada; ^19^Department of Psychiatry, University of Michigan, Ann Arbor, MI, United States; ^20^Department of Psychiatry, Queen's University, Kingston, ON, Canada; ^21^Tanz Centre for Research in Neurodegenerative Diseases University of Toronto, Toronto, ON, Canada; ^22^Department of Psychiatry, Cumming School of Medicine, University of Calgary, Calgary, AB, Canada; ^23^Ontario Brain Institute, Toronto, ON, Canada; ^24^Douglas Mental Health University Institute, McGill University, Montreal, QC, Canada; ^25^Department of Psychiatry, Dalhousie University, Halifax, NS, Canada

**Keywords:** common data elements, psychiatric comorbidity, major depressive disorder, neurological disorders, data sharing, pooled participant data, depression and anxiety, brain-code

## Abstract

The Ontario Brain Institute's “Brain-CODE” is a large-scale informatics platform designed to support the collection, storage and integration of diverse types of data across several brain disorders as a means to understand underlying causes of brain dysfunction and developing novel approaches to treatment. By providing access to aggregated datasets on participants with and without different brain disorders, Brain-CODE will facilitate analyses both within and across diseases and cover multiple brain disorders and a wide array of data, including clinical, neuroimaging, and molecular. To help achieve these goals, consensus methodology was used to identify a set of core demographic and clinical variables that should be routinely collected across all participating programs. Establishment of Common Data Elements within Brain-CODE is critical to enable a high degree of consistency in data collection across studies and thus optimize the ability of investigators to analyze pooled participant-level data within and across brain disorders. Results are also presented using selected common data elements pooled across three studies to better understand psychiatric comorbidity in neurological disease (Alzheimer's disease/amnesic mild cognitive impairment, amyotrophic lateral sclerosis, cerebrovascular disease, frontotemporal dementia, and Parkinson's disease).

## Introduction

The Ontario Brain Institute's (OBI) “Brain-CODE” informatics platform (www.braincode.ca) (1) was designed to support the collection, integration, sharing, and analysis of diverse types of patient-level data across several brain disorders, including neurodevelopmental disorders (www.pond-network.ca), cerebral palsy (www.cpnet.canchild.ca), epilepsy (www.eplink.ca), major depressive disorder (MDD, www.canbind.ca), concussion (www.connectontario.ca), and neurodegenerative/neurovascular cognitive disorders (www.ondri.ca). These programs provide an opportunity to facilitate collaboration across disorders; with pooling of data across these programs expanding the utility of the individual datasets to support cross-disease comparisons and generalizability of findings. However, combining participant-level data from multiple studies and patient populations can be challenging, as different measures are often used to assess the same constructs (2). These data must be sufficiently comparable to allow meaningful data integration, and in the absence of common demographic variables and outcome measures it is difficult to pool data from different initiatives (2). Establishment up front of a minimum set of unambiguously defined and standardized assessments across initiatives will facilitate data sharing and integration and enable meaningful cross-initiative analyses (3–6).

To help achieve this level of collaboration and data interoperability, a set of common demographic and clinical outcome measures were identified and adopted across all of the aforementioned research programs. The adoption of common data elements (CDEs) within these studies reduces the variability of data collection, and ultimately supports the secondary use of Brain-CODE data by facilitating the pooling of participant-level data across datasets (1, 3–6). The primary objective of the present study was to use the CDEs to assess psychiatric comorbidity across various neurological diseases. We present here a summary of how we determined and used the Brain-CODE CDEs, including an example using CDEs pooled across three programs to assess symptoms of depression and anxiety across neurological diseases (Alzheimer's disease/amnesic mild cognitive impairment, amyotrophic lateral sclerosis, cerebrovascular disease, frontotemporal dementia and Parkinson's disease), as well as major depressive disorder (MDD).

## Selection of CDEs

### Delphi Consensus Process

A modified Delphi survey process (7, 8) was used to identify core demographic and clinical variables to be collected across all participating programs. Researchers from the five programs were invited to an online survey hosted through the Brain-CODE portal. Participants were asked to comment and respond to statements on a 5-point Likert scale regarding the collection of demographic and clinical variables, with possible responses ranging from *Not Important* to *Very Important* (example: How important is the collection of date of birth to achieving Brain-CODE goals?) to *Do Not Recommend* to *Highly Recommend* (example: Please provide your recommendation for the GAD-7 to assess anxiety in adults across all programs) or *Disagree* to *Strongly Agree* (example: QIDS-SR is appropriate to assess depression across all programs in adults). A *Do Not Know* option was included for all questions. Participants were also provided open-ended questions to allow them to comment on their answers, provide an additional opinion about Brain-CODE CDEs beyond the specified variables, and whether additional CDEs should be considered. The results were reviewed, and anonymized aggregated ratings and comments were presented back to the participants in a follow-up survey to obtain additional opinion and clarification, as required. Participants were directed to consider the results of the previous survey in their responses.

Prior to sending out the Delphi surveys, we identified the demographic and clinical domains that would be brought forward for consensus. Following the review of research project data dictionaries, study protocols, and through interactions with program researchers, we identified the following demographic domains of relevance across programs to consider for consensus: *Sex, Date of Birth, Handedness, Ethnicity, Race, Education Level, Marital Status, Primary Language, Place of Birth, Geographic Region*, and *Height/Weight*.

For the clinical CDEs, a preliminary online survey was sent to the programs to provide opinions on the symptom domains that may be of relevance to their program and across a broad range of patient populations. The results were presented at a follow-up workshop. Following group discussion there was agreement that *psychiatric and medical comorbidity, depression, anxiety, sleep, quality of life*, and *activities of daily living* should be assessed across all programs. These domains were considered relevant across programs, as comorbid psychiatric symptoms are often reported across a broad range of patient population that can impact health (9–12). There was also agreement that when possible, the measure should be patient-reported, brief and easy to administer, widely used and validated, and available in the public domain. Based on literature and expert opinion, potential rating scales were then identified for each of the symptom domains to consider for consensus. A summary of the scales was presented to the participants in the Delphi survey with the aim of achieving consensus for a common measure for each symptom domain.

Although the threshold for consensus is arbitrary, recommended criteria for Delphi consensus generally range from 70 to 80% of agreement within two categories (8). In the present surveys, this would include ratings of *Important/Very Important, Recommend/Highly Recommend or Agree/Strongly Agree*. Consensus levels of >70% were considered, with other factors also weighted including harmonization with existing relevant databases. When consensus was not achieved, representatives of the relevant programs were asked to discuss internally and provide their recommendations. Thirty-six researchers participated in the demographic surveys and 44 in the clinical surveys. There was broad representation of opinion, including Principal Investigators (27%), Co-Principal Investigators (36%), Project Managers (27%), and Research Coordinators and Investigators (9%). The respondents indicated that the populations studied in their programs included adult (59%), child and adolescent (46%), infant (18%), and geriatric (18%).

### Demographic and Clinical CDEs Selected

The demographic and clinical CDEs that were agreed upon are shown Table 1 and are summarized in Supplementary Material. To promote uptake and standardization of the CDEs, data dictionaries for the CDEs were made available on the Brain-CODE portal and electronic case report forms were independently validated prior to data collection to ensure adherence to naming standards. These core Demographic and Clinical CDEs have been successfully implemented across participating programs (please see www.braincode.ca for most recent numbers of CDEs collected within each program).

**Table 1:** Brain-CODE demographic and clinical CDEs.

**DOMAIN**	**SUB-DOMAIN**	**Brain-CODE CDE**
Patient	Demographic	Sex, date of birth, and handedness
characteristics	Socioeconomic status (SES)	Ethnicity, marital status, occupation, and household income
Physical and	Quality of life	WHO-QoL-BREF (adult)
mental health		KINDL-R (child and adolescent)
	Activities of daily living	Sheehan disability scale (adult)
	Medical comorbidity	NINDS medical history
	Psychiatric comorbidity	BSI (adolescent and adult)
Clinical	Depression	QIDS-SR (adolescent and adult)
endpoints		RCADS (child and adolescent)
	Anxiety	GAD-7 (adolescent and adult)
		RCADS (child and adolescent)
	Sleep	PSQI (adult)
		CSHQ (adolescent and adult)

## Assessment of Psychiatric Comorbidity Across Neurological Diseases and Controls

Comorbid psychiatric symptoms are often reported across a broad range of patient populations that can impact health (9–12) and are an integral part of neurological disorders (13–15). To better understand the expression of depression and anxiety in neurological disorders, we analyzed Brain-CODE CDEs (Demographics, QIDS-SR, and GAD-7) pooled across the five neurological diseases (Alzheimer's disease/amnesic mild cognitive impairment, amyotrophic lateral sclerosis, cerebrovascular disease, frontotemporal dementia, and Parkinson's disease) and MDD.

### Method

#### Study Population and Datasets

##### Ontario Neurodegenerative Disease Research Initiative

The Ontario Neurodegenerative Disease Research Initiative (ONDRI, NCT04104373) is a multi-site prospective cohort study developed to characterize and track progression of neurodegenerative and cerebrovascular disorders (16). Cohorts include: Alzheimer's disease (AD) or amnestic single or multidomain mild cognitive impairment (MCI), amyotrophic lateral sclerosis (ALS), cerebrovascular disease (CVD), frontotemporal dementia (FTD), and Parkinson's disease (PD). These groups were included in the present study to assess the impact of depression and anxiety across neurological disorders. Please see Farhan et al. (16) and Sunderland et al. (17) for protocol details, inclusion, and exclusion criteria.

##### Canadian Biomarker Integration Network for Depression Study

The Canadian Biomarker Integration Network in Depression Study (CAN-BIND-1, NCT01655706) is a prospective multi-site study developed to identify biomarkers of antidepressant response in MDD (18, 19). The study collects imaging, clinical, and ‘omics data that will be used to build predictive models of treatment response. Cohorts include people with MDD and healthy comparison participants, both of which are included in the present analysis. Please see Lam et al. (19) for protocol details, inclusion, and exclusion criteria.

##### Brain-Eye Amyloid Memory Study

Brain-Eye Amyloid Memory Study (BEAM, NCT02524405) is a multi-site investigational study of non-invasive ocular measures in neurodegenerative disease. Cohorts include: AD/MCI, CVD, PD, and Lewy body disease and healthy comparison participants. Please see www.clinicaltrials.gov/ct2/show/NCT02524405 for protocol details. Only healthy comparison participants were included in the present analyses. In addition, although not part of the BEAM inclusion criteria, only healthy comparison participants within normal limits on both the Montreal Cognitive Assessment (Total Score ≥ 26) and the Toronto Cognitive Assessment (Sum Index score > 281) were included in the present analysis.

##### Analysis Dataset

All data from ONDRI, CAN-BIND, and BEAM were collected and deposited into the Brain-CODE database (1). The illustrative analysis dataset used in the present analysis included Brain-CODE CDE data (Demographics, QIDS-SR, GAD-7, and WHO-QoL BREF) collected at baseline in participants enrolled in ONDRI (AD/MCI, *n* = 126; ALS, *n* = 40; CVD, *n* = 161; FTD, *n* = 53; PD, *n* = 140), CAN-BIND (MDD, *n* = 211; Healthy controls, *n* = 112), and BEAM (Healthy controls, *n* = 45).

The following cohorts were included in analyses:

AD/MCI (ONDRI),
ALS (ONDRI),
CVD (ONDRI),
FTD (ONDRI),
PD (ONDRI),
MDD (CAN-BIND),
CAN-BIND healthy controls, and
BEAM health controls.

All studies were carried out in accordance with the Declaration of Helsinki and the International Council for Harmonization (ICH) of Technical Requirements for Pharmaceuticals for Human Use guidelines, and the study designs and procedures were reviewed by the appropriate ethics committees; informed consent was obtained from participants after full explanation of the nature of the procedures.

#### Study Assessments

##### Symptoms of Depression

The QIDS-SR is a 16-item self-report measure that assesses the severity of depressive symptoms based on DSM-5 criteria for a major depressive episode, with items scored on a 4-point scale from 0 to 3 (20). Scoring of the QIDS-SR converts the 16 items into 9 DSM domains (sad mood, concentration, self-criticism, suicidal ideation, interest, energy/fatigue, sleep disturbances, and changes in appetite/weight), with the total score ranging from 0 to 27. It is important to note that because of ethics concerns (challenges in providing immediate follow-up with those expressing suicidal ideation), item #12 assessing suicidality was omitted from the QIDS-SR in both ONDRI and BEAM, and was therefore removed from all analyses. This was not expected to impact the scale's ability to discriminate MDD, given that removal of suicidality in other scales [for example, PHQ-9 (with suicide item) vs. PHQ-8 (without suicide item)] does not impact the scale's psychometric properties (21).

##### Symptoms of Anxiety

The GAD-7 is a 7-item self-report measure that is used to assess the severity of generalized anxiety symptoms (22). Items rate the severity of the 7 symptoms over the past 2 week on a 4-point scale (0 = not at all, 1 = several days, 2 = more than half the days, and 3 = nearly every day), with total score ranging from 0 to 21.

#### Analyses

Demographic and clinical characteristics were calculated and compared across all cohorts; ANOVA was used for comparison of continuous variables and chi-squared for comparison of categorical variables. Analyses were performed using SPSS V26. A level of *p* < 0.05 was regarded as statistically significant. Furthermore, to assess the validity of the CDEs in assessing depression and anxiety across the different cohorts, Cronbach's alpha and item-total correlations were also calculated for the QIDS-SR and GAD-7 within each of the cohorts as a measure of the scale's internal consistency.

### Results

#### QIDS-SR and GAD-7 Internal Consistency

Table 2 shows Cronbach's alpha and item-total correlations for the QIDS-SR and GAD-7 for the ONDRI neurological disease (ND) and CAN-BIND MDD cohorts. For the QIDS-SR, alphas ranged from 0.68 (MDD) to 0.74 (FTD, PD), suggesting that although some of the items are assessing the same construct, and others may not. Notably, low item-total correlations (*r* < 0.3) were noted for the “Sleep” domain in AD/MCI, CVD, FTD, and PD, which were also questionable within the ALS (*r* = 0.33) and MDD (*r* = 0.31) cohorts. Other items identified as having low item-total correlations, included “Appetite” in AD/MCI (*r* = 0.19), PD (*r* = 0.26), which were also questionable in CVD (*r* = 0.35) and MDD (*r* = 0.30); “Concentration” in ALS (*r* = 0.24); and FTD (*r* = 0.32); Self-perception in ALS (*r* = 0.26), CVD (*r* = 0.35), FTD (*r* = 0.31). Low item-total correlations were also noted for the “psychomotor” item in ALS (*r* = 0.30) and MDD (*r* = 0.22).

**Table 2:** Internal consistency.

	**AD/MCI**	**ALS**	**CVD**	**FTD**	**PD**	**MDD**
**QIDS-SR**						
Cronbach's alpha	0.69	0.70	0.70	0.74	0.74	0.68
**Item-total**
Sleep	0.20	0.33	0.16	0.18	0.22	0.31
Sadness	0.50	0.46	0.31	0.33	0.51	0.52
Appetite	0.19	0.53	0.35	0.56	0.26	0.30
Concentration	0.45	0.24	0.51	0.32	0.56	0.46
Self-perception	0.44	0.26	0.35	0.31	0.45	0.36
Interest	0.45	0.52	0.55	0.60	0.59	0.43
Energy	0.50	0.49	0.54	0.50	0.50	0.46
Psychomotor	0.54	0.30	0.60	0.71	0.53	0.22
**GAD-7**						
Cronbach's alpha	0.88	0.85	0.89	0.83	0.92	0.84
**Item-total**
Nervous	0.65	0.70	0.78	0.78	0.78	0.66
Worry-stop	0.75	0.70	0.79	0.66	0.83	0.77
Worry too much	0.81	0.80	0.76	0.67	0.84	0.72
Relaxing	0.77	0.59	0.75	0.62	0.81	0.61
Restless	0.63	0.34	0.56	0.47	0.66	0.49
Annoyed	0.46	0.61	0.59	0.71	0.58	0.35
Afraid	0.65	0.67	0.56	0.21	0.74	0.54

For the GAD-7, alpha was good across all cohorts, ranging from 0.83 (FTD) to 0.92 (PD). Item-total correlations were also acceptable (*r* > 0.3), which the exception of the GAD-7 “afraid” item in FTD (*r* = 0.21; see Table 2).

#### Participant Demographic and Clinical Characteristics

Table 3 summarizes demographic and clinical characteristics of the ND cohorts (AD/MCI, ALS, CVD, FTD, and PD), MDD cohort and MDD- and ND-matched healthy controls. ANOVA revealed significant age differences across cohorts [*F*_(7, 888)_ = 394.77, *p* < 0.01]. No age differences were found between ONDRI ND cohorts and BEAM healthy controls or between CAN-BIND MDD and healthy controls. As expected, the ND cohorts were older than the MDD cohorts (see Table 3). Sex differences were noted across cohorts, with the proportion of female participants in the CAN-BIND cohorts representative of the sex distribution of MDD (10, 23, 24). The ONDRI ND cohorts were predominately male [ranging from 22.1% (PD) to 45.2% (AD/MCI) female], whereas the BEAM healthy control cohort was predominately female (75%). These sex differences between ONDRI ND cohorts and BEAM healthy controls may reflect differences in subject recruitment protocols, as the ONDRI protocol required participants to have a partner who knew them well during assessment, and that may have influenced sample demographics (16, 25). The participation of a knowledgeable study-partner is critical to dementia-related research to help manage study logistics (i.e., scheduling study visits, and transportation) and as informants (26). In the ONDRI study, the majority of participants had a spousal study partner and those with spousal study partners were more often male (25). Indeed, as females generally outlive males, this requirement may have resulted in biases toward recruitment of males with surviving female partners within the ONDRI study.

**Table 3:** Demographic and clinical characteristics.

**Cohort**	**AD/MCI**	**ALS**	**CVD**	**FTD**	**PD**	**MDD**	**Control (MDD)**	**Control (ND)**
**Program**	**ONDRI**	**ONDRI**	**ONDRI**	**ONDRI**	**ONDRI**	**CAN-BIND**	**CAN-BIND**	**BEAM**
*N*	126	40	161	53	140	211	112	45
Age, years ± SD	71.00 ± 8.16	62.00 ± 8.75	69.17 ± 7.41	67.89 ± 7.05	67.92 ± 6.38	35.30 ± 12.65	33.04 ± 10.74	66.73 ± 6.04
Sex, % female	45.2%	40.0%	31.7%	35.8%	22.1%	63.0%	63.4%	75.6%
Education, years ± SD	15.09 ± 2.91	13.95 ± 2.44	14.37 ± 2.85	13.66 ± 2.61	15.31 ± 2.62	14.10 ± 2.07	15.65 ± 2.25	16.31 ± 2.03
QIDS-SR total ± SD	3.93 ± 3.12	6.28 ± 3.70^*^	4.27 ± 3.11	5.36 ± 4.07^*^	5.61 ± 3.28^*^	14.92 ± 3.76^*^	2.27 ± 1.94	3.04 ± 2.11
GAD-7 total ± SD	2.30 ± 3.60	3.30 ± 4.21	2.58 ± 3.90	2.72 ± 3.71	3.24 ± 4.11	11.79 ± 5.02^*^	0.89 ± 1.73	1.04 ± 1.82

* *Significantly different than controls (p < 0.05)*.

**Figure 1:**
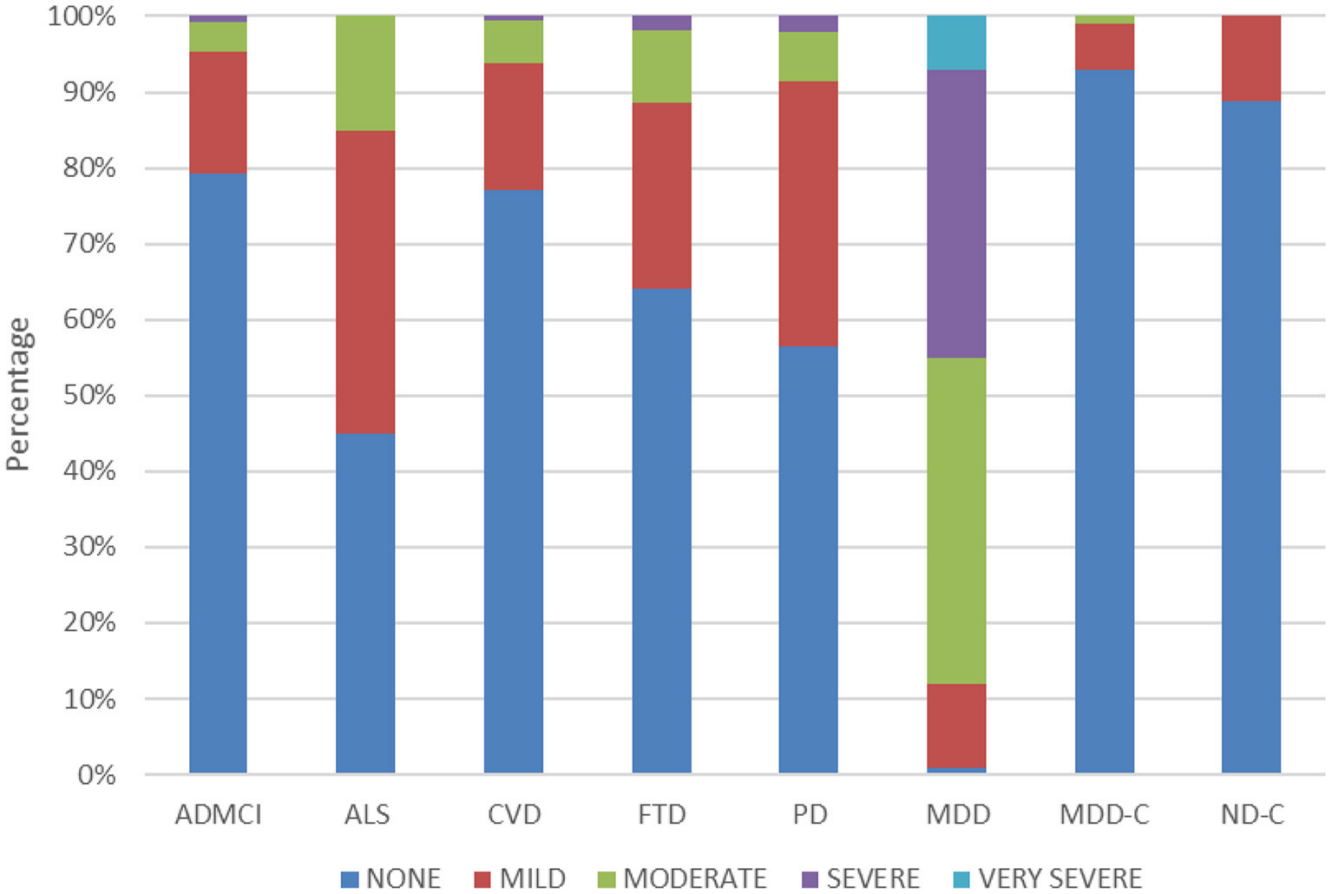
Percentage of participants reporting no (total score ≤ 5), mild (total score = 6–10), moderate (total score = 11–15), severe (total score = 16–20), and very severe (total score ≥ 21) symptoms of depression in the QIDS-SR across the cohorts.

Table 3 also shows the QIDS-SR and GAD-7 scores across cohorts. ANOVA revealed a significant difference in total QIDS-SR score across cohorts [*F*_(7, 887)_ = 261.99, *p* < 0.01]. As expected, the MDD cohort had significantly higher QIDS-SR scores (14.92 ± 3.76) than the CAN-BIND healthy controls (2.27 ± 1.94) and all ONDRI ND cohorts (3.93 ± 3.12 to 6.28 ± 3.70) (all *p* < 0.05). QIDS-SR scores for the ONDRI cohorts were significantly higher than BEAM healthy controls (3.04 ± 2.11) for ALS (6.28 ± 3.70), FTD (5.36 ± 4.07), and PD (5.61 ± 3.28) (all *p* < 0.05), but not for AD/MCI (3.93 ± 3.12, *p* = 0.77) and CVD (4.27 ± 3.11, *p* = 0.33). Figure 1 shows the percentage of participants showing different levels of depressive severity acorss cohorts. In the ALS, FTD, and PD cohorts, at least mild symptoms of depression (total score > 5) were reported by 55, 35.7, and 43.5% of participants, and least moderate symptoms of depression were reported by 15, 11.3, and 8.6% of participants, respectively. By contrast, mild or moderate levels of depression or greater were reported by 2.2 and 0% of BEAM healthy control participants, respectively (see Figure 1).

Differences were noted in total GAD-7 scores across cohorts [*F*_(7, 886)_ = 137.77, *p* < 0.01]. As expected, the MDD cohort had a significantly higher GAD-7 total score (11.79 ± 5.02) than CAN-BIND healthy controls (0.89 ± 1.73) and all ONDRI ND cohorts (2.30 ± 3.60 to 3.30 ± 4.21) (all *p* < 0.05). GAD-7 total score for the ONDRI ND cohorts, however, did not differ from BEAM healthy controls (1.04 ± 1.82; see Table 3).

## Discussion

The Ontario Brain Institute supports multidisciplinary collaborative Research Networks from across the province of Ontario and beyond, focusing on various brain conditions. These programs have generated large volumes of data that are integrated within the Brain-CODE platform to support scientific inquiry and analytics across multiple brain diseases and modalities, including clinical, imaging and molecular data (1). By providing access to participant-level data across different disorders, new hypotheses about brain disease and underlying causes will be generated, and ultimately promote new discoveries to improve patient care. To help achieve these goals, the establishment of demographic and clinical CDEs within Brain-CODE is a critical step toward enabling consistency in data collection and optimizing the ability of investigators to analyze pooled participant-level data across brain disorders. Furthermore, the Brain-CODE CDEs provide a framework to facilitate collaborations across disciplines and increase our understanding symptom expression across diseases and comorbidity.

Adopting a set of standardized assessments across different disease areas to facilitate sharing of participant-level data is a challenging endeavor that must consider the need and goals of individual research programs, since each program must select the disease-specific measures that are most relevant. As a matter of good scientific research practice, the measurements selected should be scientifically valid and justified to support specified aims. The selection of CDEs, therefore, should consider the feasibility, utility, and acceptability of outcome measures that will benefit from buy-in and cooperation from stakeholders (6), including engagement of representative researchers through participation in workshops and agreement on a set of common assessment and standards. In developing the present set of core CDEs, Delphi consensus-based methodology was used to inform and engage stakeholders, gain their input and opinion, and arrive at an agreement. By including open-ended questions and comments, suggestions and opinions were not restricted to the predefined variables, thus allowing for broadening of opinion and consideration of program-specific needs and challenges.

A key consideration was harmonizing the Brain-CODE CDEs with those of other large, centralized data repositories and CDE initiatives, as this would facilitate sharing of data among the larger research community to allow comparison of the results from one study with another. One example is the United States National Institute of Neurological Disorders and Stroke (NINDS) CDE initiative, which is developing CDEs for NINDS-supported clinical neuroscience research (27). This CDE initiative includes many disease areas that are in common with Brain-CODE, including epilepsy (28), traumatic brain injury (29) and neurodegenerative diseases (27). The Clinical Data Interchange Standards Consortium (CDISC) also provide data standards that should be considered for CDEs, including alignment with relevant Clinical Data Acquisition Standards Harmonization (CDASH)-recommended standards and guidelines for recording of demographic information (30). Indeed, alignment with existing standards should be an important consideration when designing a study to support collaborative research and data sharing, as described in the Guidelines for Data Acquisition, Quality and Curation for Observational Research Designs (DAQCORD) (31). However, it is important to recognize that although prospective standardization with existing, relevant databases is ideal to facilitate data synthesis, implementing common data collection standards with all relevant initiatives, particularly established studies and legacy data, is clearly not possible. Thus, to permit established studies or existing data to be sufficiently comparable, retrospective harmonization will also be required to define a set of core variables, including establishing conventions and equivalencies of data dictionary terms (32).

Neuropsychiatric symptoms are prevalent in neurological disorders, with rates varying anywhere from 0 to 90%, depending on the assessments used, patient samples and type of disease (33–35). Although prevalence rates across neurological disorders are difficult to ascertain as different outcome measures and criteria are often used, studies generally show prevalence rates of depression and anxiety that are higher than in the general population and negatively impact well-being and progression of disease (33–35). In the present analysis of participant-level data pooled across different studies and using standardized outcome measures, depression scores (QIDS-SR) were found to be higher in ALS, FTD, and PD cohorts, but not AD/MCI and CVD, as compared to age-matched controls (see Table 3). The mean QIDS-SR total scores for the ALS, FTD, and PD cohorts indicated overall “mild” levels of depression, as compared to the “moderate” levels observed in the MDD cohort (see Figure 1). No differences, however, were noted in GAD-7 scores in any of the ONDRI ND cohorts as compared to age-matched controls.

As part of the ONDRI protocol, individuals with “unstable psychiatric illness defined as psychosis (hallucinations or delusions), lifelong history of major depression, or untreated late-onset major depression within 90 days of the screening visit” were excluded from the study (16). As a result, individuals with more severe psychiatric symptoms or a clinical diagnosis of MDD were excluded from the ONDRI study. However, although the relatively low levels of depressive symptoms and absence of anxiety-related symptoms may in part be explained by ONDRI study selection bias, other factors may also be involved, including adaptive psychosocial adjustment and coping strategies (36, 37). This is consistent with the “disability paradox” in which persons with neurological disorders (and other chronic illnesses) adapt to their disabilities and report better well-being and quality of life than would be expected from the general population (36). Studies are currently underway using the Brain-CODE CDEs to assess psychosocial determinants of quality of life across disorders.

One of the key objectives of the CAN-BIND and ONDRI programs is the discovery biomarkers (imaging and ‘omics) to track disease and treatment response (16, 19). The present study demonstrates that some symptoms are shared across disorders, and within disorders, individual symptom profiles and phenotypes vary. Indeed, there are approximately half a million symptom profiles that meet criteria for diagnosis of MDD (38). This heterogeneity presents a challenge to traditional biomarker and drug discovery research that is based on the binary classifications of diseases, such as DSM diagnosis; and given the wide range of phenotypes within diseases, this “top-down” approach to biomarker discover is imprecise at best. There may be advantages, therefore, to also incorporate a “bottom-up” strategy to identify relevant clinical phenotypes and associated biomarkers that will refine our understand of the pathophysiology underlying disease and ultimately develop personalized treatment approaches (38, 39).

It is important that CDEs demonstrate good construct validity across the diverse populations supported by Brain-CODE. Although the clinical CDEs were selected in part because they are widely used, including in some of the disease areas currently supported in Brain-CODE, the validity of these outcome assessments across these diverse populations cannot be fully appreciated until appropriate validation is performed. In the present study, the GAD-7 showed good internal consistency across all cohorts, with Cronbach's alpha of 0.83 or greater and generally acceptable item-total correlations (see Table 3). By contrast, Cronbach's alphas for the QIDS-SR were generally lower (0.68–0.74), with item-total correlations identifying some items with poor discrimination (see Table 3). As psychiatric symptoms often overlap with symptoms observed in neurological disease, it is possible that some of the symptom assessed by the QIDS-SR and GAD-7 may be confounded with neurological disease-related symptoms (i.e., sleep, concentration and psychomotor). As validation relates not only to the instrument itself, but also how it is being used, it will be important that the measurement properties of these scales are evaluated across disease groups. These studies are currently underway, including the application of item response and Rasch measurement theory to evaluate scale performance (40).

## Data Availability Statement

The data analyzed in this study is subject to the following licenses/restrictions: participants' data used in this study are currently stored in the Brain-CODE Neuroinformatics Platform (https://www.braincode.ca/) managed by the Ontario Brain Institute. Requests to access these datasets should be directed to the Ontario Brain Institute at info@braininstitute.ca.

## Ethics Statement

The studies involving human participants were reviewed and approved by all recruitment sites in accordance with the Governance Policy of Ontario Brain Institute as well the institutional policies. The patients/participants provided their written informed consent to participate in this study.

## Author Contributions

AV: manuscript preparation, study concept and design, statistical analysis and interpretation of data, and ONDRI data management and curation. KE: study concept and design, statistical analysis, and interpretation of data. DB: statistical analysis and interpretation of data, ONDRI data management, and curation. SGE, KH, MJ, FP, and SR: CAN-BIND data management and curation. SGE, MJ, BT, BL, SL, and AS: ONDRI and BEAM data management and curation. BF, SK, RL, DPM, RM, VT, and RU: CAN-BIND recruiting site leads. SB, EF, MF, AL, MM, RS, MT, and LZ: ONDRI recruiting site leads. All authors provided critical revision of the manuscript for important intellectual content, read, and approved the final manuscript.

## Funding

This research was conducted with the support of the Ontario Brain Institute, an independent non-profit corporation, funded partially by the Ontario government. BEAM funding from Brain Canada, the Edwards Foundation and GE Healthcare for in kind support; funding was also received from Linda C. Campbell toward the BEAM study. CAN-BIND and ONDRI are Integrated Discovery Programs with support from the Ontario Brain Institute, with funding and/or in-kind support also provided by the investigators' universities and academic institutions. Additional funding for CAN-BIND was provided by CIHR, Lundbeck, Bristol-Myers Squibb, Pfizer, and Servier. The funder was not involved in the study design, collection, analysis, interpretation of data, the writing of this article or the decision to submit it for publication.

## Author Disclaimer

The opinions, results, and conclusions are those of the authors and no endorsement by the Ontario Brain Institute is intended or should be inferred.

## Conflict of Interest

Author RL has received honoraria for ad hoc speaking or advising/consulting, or received research funds, from Allergan, Asia-Pacific Economic Cooperation, BC Leading Edge Foundation, Canadian Institutes of Health Research, Canadian Network for Mood and Anxiety Treatments, Healthy Minds Canada, Janssen, Lundbeck, Lundbeck Institute, Michael Smith Foundation for Health Research, MITACS, Myriad Neuroscience, Ontario Brain Institute, Otsuka, Unity Health, Viatris, and VGH-UBCH Foundation. Author RM has received consulting and speaking honoraria from AbbVie, Allergan, Eisai, Janssen, KYE, Lallemand, Lundbeck, Neonmind, Otsuka, and Sunovion, and research grants from CAN-BIND, CIHR, Janssen, Lallemand, Lundbeck, Nubiyota, OBI, and OMHF. Author SP has received honoraria for consulting or research funds from Assurex (Myriad), Sage, Otsuka, Takeda, Janssen, Aifred, Mensante, Canadian Institutes for Health Research, Ontario Brain Institute, and the Flinn Foundation. Author SB has received honoraria for ad hoc advising for Hoffman LaRoche, and Biogen Canada, and speaker honoraria from Biogen Canada, and in-kind support from Lilly-Avid and GEHealthcare. The remaining authors declare that the research was conducted in the absence of any commercial or financial relationships that could be construed as a potential conflict of interest.

## Publisher's Note

All claims expressed in this article are solely those of the authors and do not necessarily represent those of their affiliated organizations, or those of the publisher, the editors and the reviewers. Any product that may be evaluated in this article, or claim that may be made by its manufacturer, is not guaranteed or endorsed by the publisher.

## References

[1] Vaccarino AL Dharsee M Strother S Aldridge D Arnott SR Behan B. Brain-CODE: a secure neuroinformatics platform for management, federation, sharing and analysis of multi-dimensional neuroscience data. Front Neuroinform. (2018) 12:28. 10.3389/fninf.2018.00028 29875648 PMC5974337

[2] Siddique J de Chavez PJ Howe G Cruden G Brown CH. Limitations in using multiple imputation to harmonize individual participant data for meta-analysis. Prev Sci. (2018) 19(Suppl. 1):95–108. 10.1007/s11121-017-0760-x 28243827 PMC5572105

[3] Cohen MZ Thompson CB Yates B Zimmerman L Pullen CH. Implementing common data elements across studies to advance research. Nurs Outlook. (2015) 63:181–8. 10.1016/j.outlook.2014.11.006 25771192 PMC4361774

[4] Gliklich RE Castro M Leavy MB Press VG Barochia A Carroll CL. Harmonized outcome measures for use in asthma patient registries and clinical practice. J Allergy Clin Immunol. (2019) 144:671–81.e1. 10.1016/j.jaci.2019.02.025 30857981

[5] Redeker NS Anderson R Bakken S Corwin E Docherty S Dorsey SG. Advancing symptom science through use of common data elements. J Nurs Scholarsh. (2015) 47:379–88. 10.1111/jnu.12155 26250061 PMC4618317

[6] Sheehan J Hirschfeld S Foster E Ghitza U Goetz K Karpinski J. Improving the value of clinical research through the use of Common Data Elements. Clin Trials. (2016) 13:671–6. 10.1177/1740774516653238 27311638 PMC5133155

[7] Dalkey NC Helmer O. An experimental application of the Delphi method to the use of experts. Manag Sci. (1963) 9:458–67. 10.1287/mnsc.9.3.458

[8] Hsu CC Standford BA. The Delphi technique: making sense of consensus. Pract Assess Res Eval. (2007) 12:1–8. 10.7275/pdz9-th90

[9] Gadermann AM Alonso J Vilagut G Zaslavsky AM Kessler RC. Comorbidity and disease burden in the National Comorbidity Survey Replication (NCS-R). Depress Anxiety. (2012) 29:797–806. 10.1002/da.21924 22585578 PMC4005614

[10] Kessler RC McGonagle KA Zhao S Nelson CB Hughes M Eshleman S. Lifetime and 12-month prevalence of DSM-III-R psychiatric disorders in the United States. Results from the National Comorbidity Survey. Arch Gen Psychiatry. (1994) 51:8–19. 10.1001/archpsyc.1994.03950010008002 8279933

[11] Kessler RC Zhao S Blazer DG Swartz M. Prevalence, correlates, and course of minor depression and major depression in the National Comorbidity Survey. J Affect Disord. (1997) 45:19–30. 10.1016/S0165-0327(97)00056-6 9268772

[12] Kessler RC Berglund P Demler O Jin R Koretz D Merikangas KR. The epidemiology of major depressive disorder: results from the National Comorbidity Survey Replication (NCS-R). JAMA. (2003) 289:3095–105. 10.1001/jama.289.23.3095 12813115

[13] Galts CPC Bettio LEB Jewett DC Yang CC Brocardo PS Rodrigues ALS. Depression in neurodegenerative diseases: common mechanisms and current treatment options. Neurosci Biobehav Rev. (2019) 102:56–84. 10.1016/j.neubiorev.2019.04.002 30995512

[14] Prisnie JC Sajobi TT Wang M Patten SB Fiest KM Bulloch AGM. Effects of depression and anxiety on quality of life in five common neurological disorders. Gen Hosp Psychiatry. (2018) 52:58–63. 10.1016/j.genhosppsych.2018.03.009 29684713

[15] Roos E Mariosa D Ingre C Lundholm C Wirdefeldt K Roos PM. Depression in amyotrophic lateral sclerosis. Neurology. (2016) 86:2271–7. 10.1212/WNL.0000000000002671 27164661 PMC4909561

[16] Farhan SM Bartha R Black SE Corbett D Finger E Freedman M. The Ontario Neurodegenerative Disease Research Initiative (ONDRI). Can J Neurol Sci. (2017) 44:196–202. 10.1017/cjn.2016.415 28003035

[17] Sunderland KM Beaton D Fraser J Kwan D McLaughlin PM Montero-Odasso M. The utility of multivariate outlier detection techniques for data quality evaluation in large studies: an application within the ONDRI project. BMC Med Res Methodol. (2019) 19:102. 10.1186/s12874-019-0737-5 31092212 PMC6521365

[18] Kennedy SH Lam RW Rotzinger S Milev RV Blier P Downar J. Symptomatic and functional outcomes and early prediction of response to escitalopram monotherapy and sequential adjunctive aripiprazole therapy in patients with major depressive disorder: a CAN-BIND-1 report. J Clin Psychiatry. (2019) 80:18m12202. 10.4088/JCP.18m12202 30840787

[19] Lam RW Milev R Rotzinger S Andreazza AC Blier P Brenner C. Discovering biomarkers for antidepressant response: protocol from the Canadian biomarker integration network in depression (CAN-BIND) and clinical characteristics of the first patient cohort. BMC Psychiatry. (2016) 16:105. 10.1186/s12888-016-0785-x 27084692 PMC4833905

[20] Rush AJ Trivedi MH Ibrahim HM Carmody TJ Arnow B Klein DN. The 16-Item Quick Inventory of Depressive Symptomatology (QIDS), clinician rating (QIDS-C), and self-report (QIDS-SR): a psychometric evaluation in patients with chronic major depression. Biol Psychiatry. (2003) 54:573–83. 10.1016/S0006-3223(02)01866-8 12946886

[21] Shin C Lee SH Han KM Yoon HK Han C. Comparison of the usefulness of the PHQ-8 and PHQ-9 for screening for major depressive disorder: analysis of psychiatric outpatient data. Psychiatry Investig. (2019) 16:300–5. 10.30773/pi.2019.02.01 31042692 PMC6504773

[22] Spitzer RL Kroenke K Williams JB Löwe B. A brief measure for assessing generalized anxiety disorder: the GAD-7. Arch Intern Med. (2006) 166:1092–7. 10.1001/archinte.166.10.1092 16717171

[23] Gater R Tansella M Korten A Tiemens BG Mavreas VG Olatawura MO. Sex differences in the prevalence and detection of depressive and anxiety disorders in general health care settings: report from the World Health Organization Collaborative Study on Psychological Problems in General Health Care. Arch Gen Psychiatry. (1998) 55:405–13. 10.1001/archpsyc.55.5.405 9596043

[24] Salk RH Hyde JS Abramson LY. Gender differences in depression in representative national samples: meta-analyses of diagnoses and symptoms. Psychol Bull. (2017) 143:783–822. 10.1037/bul0000102 28447828 PMC5532074

[25] Sunderland KM Beaton D Arnott SR Kleinstiver P Kwan D Lawrence-Dewar JM Ramirez J. The Ontario neurodegenerative disease research initiative. medRXiv [Preprint]. (2020). 10.1101/2020.07.30.20165456. Available online at: https://www.medrxiv.org/content/10.1101/2020.07.30.20165456v1.full.pdf (accessed January 20, 2021).

[26] Black BS Taylor H Rabins PV Karlawish J. Researchers' perspectives on the role of study partners in dementia research. Int Psychogeriatr. (2014) 26:1649–57. 10.1017/S1041610214001203 24990196 PMC4349344

[27] Grinnon ST Miller K Marler JR Lu Y Stout A. National Institute of Neurological Disorders and Stroke Common Data Element Project - approach and methods. Clin Trials. (2012) 9:322–9. 10.1177/1740774512438980 22371630 PMC3513359

[28] Loring DW Lowenstein DH Barbaro NM Fureman BE Odenkirchen J Jacobs MP. Common data elements in epilepsy research: development and implementation of the NINDS epilepsy CDE project. Epilepsia. (2011) 52:1186–91. 10.1111/j.1528-1167.2011.03018.x 21426327 PMC3535455

[29] Thurmond VA Hicks R Gleason T Miller AC Szuflita N Orman J. Advancing integrated research in psychological health and traumatic brain injury: common data elements. Arch Phys Med Rehabil. (2010) 91:1633–6. 10.1016/j.apmr.2010.06.034 21044705

[30] Gaddale JR. Clinical data acquisition standards harmonization importance and benefits in clinical data management. Perspect Clin Res. (2015) 6:179–83. 10.4103/2229-3485.167101 26623387 PMC4640009

[31] Ercole A Brinck V George P Hicks R Huijben J Jarrett M. Guidelines for data acquisition, quality and curation for observational research designs (DAQCORD). J Clin Transl Sci. (2020) 4:354–9. 10.1017/cts.2020.24 33244417 PMC7681114

[32] Fortier I Doiron D Little J Ferretti V L'Heureux F Stolk RP. International Harmonization Initiative, Is rigorous retrospective harmonization possible? Application of the DataSHaPER approach across 53 large studies. Int J Epidemiol. (2011) 40:1314–28. 10.1093/ije/dyr106 21804097 PMC3204208

[33] Timmer MHM van Beek MHCT Bloem BR Esselink RAJ. What a neurologist should know about depression in Parkinson's disease. Pract Neurol. (2017) 17:359–68. 10.1136/practneurol-2017-001650 28739866

[34] Lyketsos CG Carrillo MC Ryan JM Khachaturian AS Trzepacz P Amatniek J. Neuropsychiatric symptoms in Alzheimer's disease. Alzheimers Dement. (2011) 7:532–9. 10.1016/j.jalz.2011.05.2410 21889116 PMC3299979

[35] Nagy A Schrag A. Neuropsychiatric aspects of Parkinson's disease. J Neural Transm (Vienna). (2019) 126:889–96. 10.1007/s00702-019-02019-7 31144104

[36] Albrecht GL Devlieger PJ. The disability paradox: high quality of life against all odds. Soc Sci Med. (1999) 48:977–88. 10.1016/S0277-9536(98)00411-0 10390038

[37] Benbrika S Desgranges B Eustache F Viader F. Cognitive, emotional and psychological manifestations in amyotrophic lateral sclerosis at baseline and overtime: a review. Front Neurosci. (2019) 13:951. 10.3389/fnins.2019.00951 31551700 PMC6746914

[38] Barron DS Baker JT Budde KS Bzdok D Eickhoff SB Friston KJ. Decision models and technology can help psychiatry develop biomarkers. Front. Psychiatry. (2021) 12:706655. 10.3389/fpsyt.2021.706655 34566711 PMC8458705

[39] Caspani G Turecki G Lam RW Milev RV Frey BN MacQueen GM. Metabolomic signatures associated with depression and predictors of antidepressant response in humans: a CAN-BIND-1 report. Commun Biol. (2021) 4:903. 10.1038/s42003-021-02421-6 34294869 PMC8298446

[40] Vaccarino AL Kalali AH Blier P Gilbert Evans S Engelhardt N. THE DEPRESSION INVENTORY DEVELOPMENT SCALE: assessment of psychometric properties using classical and modern measurement theory in a CAN-BIND trial. Innov Clin Neurosci. (2020) 17:30–40. 10.1037/t79922-000 33520402 PMC7839654

