# Supplementary material for: Common Data Elements to Facilitate Sharing and Re-use of Participant-Level Data: Assessment of Psychiatric Comorbidity Across Brain Disorders
Source: Front Psychiatry. 2022 Feb 7;13:816465. doi: 10.3389/fpsyt.2022.816465 (PMC8859302; doi:10.3389/fpsyt.2022.816465)
Supplement: Supplementary file 1 [file Data_Sheet_1.docx]

**Delphi consensus outcomes**

1. **Demographic CDEs**

**Sex**

There was consensus that the subject’s phenotypic sex should be recorded across programs (>90% agreement) to support analyses based on population stratification and reporting in peer-reviewed publications (1).

**Date of Birth (DOB)**

Based on the results from Survey 1, there was general agreement that recording of Date of Birth (DOB) is relevant to achieving the goals of Brain-CODE (>80% agreement). Participants cited a number of reasons to support the inclusion of DOB, including the ability to publish findings: “reporting on the mean age and age range of your study subjects is standard for all peer-reviewed manuscripts…” Indeed, the International Committee of Medical Journal Editors (2013) requires that “where scientifically appropriate, analyses of the data by such variables as age and sex should be included.” Other reasons participants cited to support inclusion of DOB included the ability to define study inclusion criteria: “To support age range inclusion … Date of birth will establish the eligibility criteria to participate in the project,” and study-related factors: “DOB is critical for measuring clinical parameters such as time-to-onset … we collect neuropsychological and neuroimaging outcome measures and we need to control for age-related changes in cognition and brain tissue.” However, although there was clear overall support for the inclusion of DOB, differences were noted with respect to the study population. As indicated by participants, full DOB was seen as less critical in studies involving adults: “note I put somewhat important for adults and seniors in my own project as I consider year of birth adequate for them rather than full DOB … year of birth is most important for our study (Adults). The exact date is not that important.”

These results were summarized and presented in Survey 2. The participants’ opinions were queried regarding the collection of partial DOB (month and year or year only). As compared to the collection of full DOB, there was less support for collection of partial DOB with 58% and 67% agreement that recording of month and year was sufficient in children/adolescents and adults, respectively. There was little or no support for collection of the year only (0% for children/adolescents and 25% for adults). Participant’s comments re-iterated the distinctions with respect to study populations: *“Month is important for infants and possibly children, but year should suffice for adult and geriatric populations… in work with adults, we typically need age information for many reasons (comparison with normative data, time-to-onset, duration of illness) and this is nearly 100% captured with month and year and likely captured for 80% of all situations with year alone…”* None-the-less, there was support for capturing full DOB over partial DOB, as full DOB would provide the most detailed information required for calculation of age: *“Having the full DOB allows for the calculation of age at the time of study participation with no margin of error”* and could potentially be used for Brain-CODE linkage purposes: *“Matching subjects' data with their date of birth is a way to corroborate the accuracy of the Health Card ID… DOB is a key indicator that helps to differentiate one subject from another.”* Indeed, majority of Ontarians use a unique identifier to access the healthcare system, the Ontario Health Insurance Plan (OHIP) number, and is being used to link participant-level data within Brain-CODE to external administrative databases without revealing participant identity (2). It is also important to note that because full DOB is considered a personal identiﬁer, it is subject to research ethics oversight and adherence to Personal Health Information Protection Act (PHIPA) (<http://www.e-laws.gov.on.ca/html/statutes/english/elaws_statutes_04p03_e.htm>). The format for full DOB was recorded to provide unambiguous dates, as recommended by Clinical Data Acquisition Standards Harmonization (CDASH) (3).

**Handedness**

In survey 1, there was support for collecting handedness, with 64% agreeing that handedness should be recorded across programs (Survey 1). The primary reason for support was that handedness is routinely collected in brain imaging studies, as it reflects cerebral lateralization (Knecht et al., 2000). As imaging data (including magnetic resonance imaging) are collected by participating programs, participants were asked for additional feedback in Survey 2, considering the collection of imaging data as part of the Brain-CODE platform. In Survey 2, support for the inclusion of handedness increased (73% agreement) with participants acknowledging the importance of collecting handedness for imaging results: *“important to imaging studies… am willing to defer to imaging researchers who identify it as important… variable can have an impact on language lateralization and imaging result ...”* Thus, considering that handedness reflects cerebral lateralization (4) and is necessarily recorded for brain imaging studies, it was recommended that handedness be recorded across all participating programs. The format for recording of handedness was harmonized with NINDS CDE definitions.

**Race and Ethnicity**

In Survey 1, 74% agreed that ethnicity should be recorded across all programs, whereas only 55% supported the collection of race. Support for collection of ethnicity included:  "… ethnicity with its linkage to cultural factors has a more likely impact on cognition than race… Given the emerging understanding of genetic associations… and metabolism of drugs, this is important information to have… Certain genetic forms of epilepsy and certain responses to anticonvulsant drugs relate to race/ethnicity.” By contrast, participant comments did not support collection of race data: “Why would we ask for 'race' as opposed to 'ethnicity'? ... Collecting ethnicity already which is sufficient… Self-reported 'race' has more to do with societal concepts of skin colour than it does with real genetic heritage.”

It should be noted that although race and ethnicity are core NINDS CDEs, harmonizing with US standards was not recommended, given differences in Canadian and US census-based categorizations. As there was interest in collecting ethnicity data as a variable in epidemiological and genetic research, it was recommended that self-identified ethnicity should be recorded using Canadian census-based, multiple-choice categories (5). For pediatric studies, parental ethnicity was also recorded. This multi-ethnic classification will ensure broad representation across diverse and often underrepresented populations, which may be particularly important in the conduct of genome-wide association studies to support discovery and reduce disparities across ethnic groups (6)

**Social Economic Status (SES)**

Although not originally identified as a demographic variable to consider, when asked if there were any additional demographic variables that may be important in achieving Brain-CODE goals, there was support for inclusion of SES: “we need to establish a socioeconomic status index for use in some analyses … Parental SES can be an important predictor in studies with developmental disorders.” SES includes assessment of education, marital status, occupation and income. In Survey 1, there was clear support for including **Education Level**, with over 80% of participants agreeing that education level should be collected across programs: “Education is key for interpreting cognitive tests results and has been used as an important proxy variable in studies of cognitive/cerebral reserve … level of education is correlated with brain and cognitive reserve capacity … Education could moderate response to treatment … Education has a significant impact on brain development and degeneration.” One participant pointed out that “it is also a proxy for socio economic status.”

By contrast, support for the inclusion of Marital Status was somewhat mixed (<50% *agreement)*, with some supporting inclusion: “Standard demographics that is expected to be reported… It is likely that marital status will have an impact on certain aspects/determinants of health… Marital status could somewhat moderate response to treatment… Marriage status affects men and women differentially and has been shown to impact health,” whereas others did not: “Marital status is irrelevant in modern society.”

To further address this, Survey 2 participants were asked opinion regarding collection of SES. There was support for the inclusion of SES over education level only: “*while SES is often correlated with level of education it is not synonymous with SES… we lose a lot of information about the participant by excluding SES.”* Given the support for the collection of SES and considering that social status is a core NINDS Demographic CDE, it was recommended that SES should be recorded across all participating programs. The recording of Education, Marital, Employment and Job classification was harmonized with the NINDS CDE definitions.

**Variables not recommended**

Primary Language, Place of Birth, Geographic Region and Height/Weight were not recommended as core demographic variables. Place of Birth, Geographic Region and Height/Weight were endorsed by <50% of participants in Survey 1 and thus were not advanced. Although there was some support for recording Primary Language in Survey 1, with 50% and 53% agreeing it was “Important/Very Important” to record in children/adolescents and adults, respectively, support was primarily related to subject enrollment and comprehension of study protocols*: “We require that the subjects are able to read and understand English but it does not matter to us what their first or primary language is… English reading fluency required or not eligible… Only English speaking participants are being enrolled due to the behavioural therapy only being available in English…”* When this information was presented back to the participant in Survey 2, there was no clear support for inclusion with 38% and 50% “Strongly Agreeing/Agreeing” that primary language should be routinely recorded in children/adolescents and adults, respectively. As in Survey 1, support was related to subject enrollment and comprehension of study protocols: *“Only important that they read and understand English for the purposes of informed consent and full participation in the study protocols.”*

1. **Clinical CDEs**

**Psychiatric and Medical Comorbidity**

At the workshop, there was agreement to use the NINDS Medical History form to assess medical comorbidity. When surveyed regarding instruments to assess comorbid psychiatric symptoms and asked to choose among the available instruments, both the Symptom Checklist-90-R (SCL-90-R) and Brief Symptom Inventory (BSI) were endorsed. As the BSI covers the same domains as the SCL-90 and requires less time to administer, the BSI was recommended. The 53-item BSI covers 9 primary symptom domains: somatization, obsessive-compulsive, interpersonal sensitivity, depression, anxiety, hostility, phobic anxiety, paranoid ideation, and psychoticism (7).

**Depression and Anxiety**

For adults and adolescents, the Quick Inventory of Depressive Symptomatology-Self-report (QIDS-SR) was recommended for assessment of depression with 90% agreement, and the Generalized Anxiety Disorder-7 (GAD-7) to assess anxiety with 83% agreement. The QIDS-SR is a 16 item self-report measure that assesses the severity of depressive symptoms based on DSM-5 criteria for major depressive episodes (8). The GAD-7 is a 7 item self-report measure that is used to assess the severity of generalized anxiety symptoms (9). Although consensus was not achieved for assessment of depression and anxiety in children, following further internal evaluation by the relevant programs (CPNET, POND and EPLINK), the Revised Children's Anxiety and Depression Scale (RCADS) was recommended for children and adolescents, a 47-item self-reported measure that assesses separation anxiety disorder, social phobia, generalized anxiety disorder, panic disorder, obsessive compulsive disorder, and major depressive disorder (10).

**Sleep Disturbances**

The Pittsburgh Sleep Quality Index (PSQI) was recommended to assess sleep in adults and adolescents with 88% agreement. The PSQI is a self-rated questionnaire that primarily assesses nighttime sleep problems (11). Although consensus was not achieved by the survey for assessment of sleep in children, following further internal evaluation by the relevant programs, the Children’s Sleep Habit Questionnaire (CSHQ) was recommended. CSHQ is a 45-item scale designed to screen for the most common sleep problems in children and evaluates the child’s sleep based on behavior within eight different subscales: bedtime resistance, sleep-onset delay, sleep duration, sleep anxiety, night awakenings, parasomnias, sleep-disordered breathing, and daytime sleepiness (12).

**Quality of Life and Activities of Daily Living**

At the Clinical CDE Workshop, there was general agreement to use the World Health Organization Quality of Life Short Version (13), a 26 item self-report questionnaire that assesses 4 domains of quality of life: physical health, psychological health, social relationship and environment (WHOQOL Group, 1998). Although consensus was not achieved by the survey for assessment of QoL in children and adolescents, following further internal evaluation by the relevant programs, the KINDL-R was recommended. The KINDL-R is a generic instrument for assessing Health-Related Quality of Life in children and adolescents, with 3 different versions of the instrument suitable for different age groups and developmental stages (14). With respect to activities of daily living (ADLs), it was recognized that the specific ADL scale used may vary given the population/disability studied. Therefore, although the Sheehan Disability Scale (15) was recommended, there was agreement that additional disease-specific scales should be used, as appropriate.

**References**

1. International Committee of Medical Journals. (2013), Recommendations for the Conduct, Reporting, Editing, and Publication of Scholarly Work in Medical Journals, Available online at: [icmje-recommendations.pdf](http://www.icmje.org/icmje-recommendations.pdf)
2. Gee, T., Behan, B., Lefaivre, S., Azimaee, M., Dharsee, M., El Emam, K., Yang, J., Vaccarino, A., Evans, K., Victor, J. C. and Theriault, E. (2018) “Designing and Implementing a Privacy Preserving Record Linkage Protocol”, International Journal of Population Data Science, 3(4). doi: 10.23889/ijpds.v3i4.831.
3. CDISC CDASH core and domain teams. (2008). Clinical Data Acquisition Standards Harmonization (CDASH). Available online at: http://www.cdisc.org/stuff/contentmgr/files/0/9b32bc345908ac4c31ce72b529a3d995/misc/cdash_std_1_0_2008_10_01.pdf
4. Knecht, S., Dräger, B., Deppe, M., Bobe, L., Lohmann, H., Flöel, A., et al. (2000). Handedness and hemispheric language dominance in healthy humans. *Brain* 123, 2512-2518
5. Ontario Health Study. (2020), Baseline 1 Questionnaire, Available online at: [OHS Baseline Questionnaire (ontariohealthstudy.ca)](https://www.ontariohealthstudy.ca/wp-content/uploads/2020/08/OHS-Baseline-1-Qx-final-version-withToC-clean.pdf)
6. Wojcik, G.L., Graff, M., Nishimura, K.K. *et al.* Genetic analyses of diverse populations improves discovery for complex traits. *Nature* **570,**514–518 (2019). https://doi.org/10.1038/s41586-019-1310-4
7. Derogatis LR, Melisaratos N. The Brief Symptom Inventory: an introductory report. Psychol Med. 1983 Aug;13(3):595-605. PMID: 6622612.
8. Rush AJ, Trivedi MH, Ibrahim HM, Carmody TJ, Arnow B, Klein DN, Markowitz JC, Ninan PT, Kornstein S, Manber R, Thase ME, Kocsis JH, Keller MB. The 16-Item Quick Inventory of Depressive Symptomatology (QIDS), clinician rating (QIDS-C), and self-report (QIDS-SR): a psychometric evaluation in patients with chronic major depression. Biol Psychiatry. 2003 Sep 1;54(5):573-83. doi: 10.1016/s0006-3223(02)01866-8. Erratum in: Biol Psychiatry. 2003 Sep 1;54(5):585. PMID: 12946886.
9. Spitzer RL, Kroenke K, Williams JB, Löwe B. A brief measure for assessing generalized anxiety disorder: the GAD-7. Arch Intern Med. 2006 May 22;166(10):1092-7. doi: 10.1001/archinte.166.10.1092. PMID: 16717171.
10. Chorpita BF, Moffitt CE, Gray J. Psychometric properties of the Revised Child Anxiety and Depression Scale in a clinical sample. Behav Res Ther. 2005 Mar;43(3):309-22. doi: 10.1016/j.brat.2004.02.004. PMID: 15680928.
11. Buysse DJ, Reynolds CF 3rd, Monk TH, Berman SR, Kupfer DJ. The Pittsburgh Sleep Quality Index: a new instrument for psychiatric practice and research. Psychiatry Res. 1989 May;28(2):193-213. doi: 10.1016/0165-1781(89)90047-4. PMID: 2748771.
12. Owens JA, Spirito A, McGuinn M. The Children's Sleep Habits Questionnaire (CSHQ): psychometric properties of a survey instrument for school-aged children. Sleep. 2000 Dec 15;23(8):1043-51. PMID: 11145319.
13. Development of the World Health Organization WHOQOL-BREF quality of life assessment. The WHOQOL Group. Psychol Med. 1998 May;28(3):551-8. doi: 10.1017/s0033291798006667. PMID: 9626712.
14. Ravens-Sieberer U, Bullinger M. Assessing health-related quality of life in chronically ill children with the German KINDL: first psychometric and content analytical results. Qual Life Res. 1998 Jul;7(5):399-407. doi: 10.1023/a:1008853819715. PMID: 9691720.
15. Sheehan DV, Harnett-Sheehan K, Raj BA. The measurement of disability. Int Clin Psychopharmacol. 1996 Jun;11 Suppl 3:89-95. doi: 10.1097/00004850-199606003-00015. PMID: 8923116.
